# Central Texas Medical Association, Etc.

**Published:** 1894-06

**Authors:** 


**Central Texas Medical Association** will hold its semi-annual session in Waco, July 10th and 11th prox. Dr. J. M. Frazier, late of Morgan, now of Belton, is President, and Dr. H. S. Taylor, Secretary.

Dr. O. I. Halbert, the county physician, will have a paper on Tuberculosis.

Dr. W. R. Blailock, a paper on Asepsis and Antisepsis; Dr. R.

C. Nettles, a paper on Contagion; Dr. D. Parker, on Organizing the Profession; Dr. Hale, on Pelvic Cellulitis; Dr. E. C. Gordon, on Nephritis; Dr. Howard, on the Hypodemic Syringe; Dr. M. D. Knox, on Valvular Disease of the Heart. It will be a most interesting meeting, and all good doctors should attend.

---

**The Transactions of the Pan-American Medical Congress.**—The proceedings of the first Pan-American Medical Congress were compiled by the Secretary-General, Dr. Charles A. L. Reed, and transmitted to the Department of State in November, 1893. By recent joint resolution in the Senate and House of Representatives, the manuscript was transmitted to Congress, and a concurrent resolution has been adopted directing a public printer to print the same. The manuscript is now in the office of the public printer, and will be put to press at once, under the supervision of the Editorial Committee, of which Prof. John Guiteras, of Philadelphia, is the chairman.

---

**Blue Noses or Noses "Blew."**—"Let me feel your head," said the itinerant phrenologist to Suggs. "No," said Suggs, "you may feel my hat." (The hat was felt.)

Now, when one blows his nose is it a "blew" nose? (When we have a fierce cold wind in Texas they say it is a "blue norther," because the wind "blew" so hard presumably); "the wind blew a hurricane," etc.

At the druggists' convention (State Pharmacal Association) in Austin last month, a "query" was propounded: "How best can the red color of the nose caused by drinking, be removed?" One delegate exclaimed, "Oh, the nose be blowed! what are we here for?" And "blowed" or "blewed" it was, for another delegate settled the question by offering, as answer to the query, "let him keep on drinking until the nose gets blue." (That was a terrible blast.)

Another query was, "why can a man in San Antonio (dry climate) drink fifty consecutive drinks, regulation size, without feeling the effect of it; while in Galveston twenty-five drinks will make him as drunk as a biled owl?" They all gave it up,—and one gentleman of an inquisitive turn, wanted to know if such was a fact? Referred to Professor Kennedy for investigation and report at the next meeting.

